# Imaging Biomarkers in Translational Small Animal Models

**DOI:** 10.1155/2019/9469041

**Published:** 2019-02-04

**Authors:** Pablo Aguiar, Anxo Fernández-Ferreiro, Filippo Galli, Charalampos Tsoumpas

**Affiliations:** ^1^Molecular Imaging Group, Nuclear Medicine Dept., Health Research Institute of Santiago de Compostela (IDIS), 15706 Santiago de Compostela, Spain; ^2^Molecular Imaging and Medical Physics Group, Radiology Dept., University of Santiago de Compostela, Santiago de Compostela, Spain; ^3^Clinical Pharmacology Group, Health Research Institute of Santiago de Compostela (IDIS), 15706 Santiago de Compostela, Spain; ^4^Nuclear Medicine Unit, Faculty of Medicine and Psychology, Department of Medical-Surgical Sciences and Translational Medicine, “Sapienza” University, Roma, Italy; ^5^Biomedical Imaging Science Department, Leeds Institute of Cardiovascular and Metabolic Medicine, School of Medicine, University of Leeds, Leeds, UK; ^6^Research & Development, Invicro, London, UK; ^7^Translational Molecular Imaging Institute, Mount Sinai, New York, NY, USA

Preclinical molecular imaging has become an essential tool in translating the scientific understanding that has been gained by studying and developing the animal models of multiple human diseases to the clinic [1]. Today, several of the current clinical imaging techniques are already available for use in preclinical imaging models. It has allowed that same parameters can be derived from preclinical and clinical images, which has meant a swift translational trajectory for many clinical trials in oncology [2], neurology [3] or inflammation [4], or cardiology [5]. These parameters can be considered as imaging biomarkers because they represent objective physical and biological characteristics of tissues, tumours, or organs, and they can help to identify normal or pathogenic processes and treatment response. Nevertheless, the use of preclinical molecular imaging is still limited due to the lack of efficient and standardized methods for extracting accurate and reproducible imaging biomarkers for each particular disease model. The translational benefit of preclinical molecular imaging will substantially increase by improving the reliability of the collected data [6]. In this regard, a key challenge in translational molecular imaging is to define appropriate imaging biomarkers for each disease for prediction of therapeutic outcome and follow-up of new treatments. This will help in swift and successful translation of studies from small animal models to patients and consequently reduce the cost of drug discovery portfolio. This special issue focuses on the recent advances in quantitative imaging biomarkers that can be used for translational research. In particular, it promotes the discussion of the various methods that benefit from the use of markers derived from preclinical imaging techniques that can be directly transferred to clinical imaging.

This special issue contains both review (2) and original articles (8), and its focus is to provide insights into the methodologies to investigate new imaging biomarkers in translational small animal models. An open call for papers was announced in December 2017, and the submission deadline was in August 2018. In total, 20 articles were submitted, and 10 articles were accepted for publication. In terms of imaging modality, all articles used magnetic resonance imaging (MRI) (7 articles) and/or positron emission tomography (PET) (7 articles), of which 3 articles utilised both MRI and PET. Only one of the review articles included other imaging techniques, but even in that case, PET and MRI were the predominant imaging techniques. Therefore, it seems that both PET and MRI are efficient research tools in the domain of translational preclinical imaging. In terms of disease animal models, the clinical conditions were mainly oncology (brain, breast, and pancreatic cancer) and cardiovascular diseases (cardiovascular dysfunction and atherosclerosis) and other clinical fields such as radiotherapy (neutron capture and high energy photons). Obviously, the articles included in this special issue represent only a small part of the uses of translational molecular imaging in animal models, but we believe that they can be representative in relative terms. The predominance of PET and MRI could be interpreted as the consequence of their higher translational potential if compared with optical imaging or ultrasound.

In brief, the published papers on oncology show biomarkers for monitoring therapies such as radiation treatment and its side effects. S. De Bruycker et al. reviewed the different approaches to generate hypoxic in vivo cancer models directly related to PET imaging. N. Kovács et al. in their original paper performed a study to monitor the dose-limiting organs in patients undergoing radiotherapy. They report that conventional SUV values derived from brain PET and apparent diffusion coefficients from DWI can be considered as biomarkers for the follow-up of the health status after radiation therapy.

Among the other research articles, some were focused on new tracers and contrast agents. A. Leftin and J. A. Koutcher showed that tumour-associated macrophages can be accurately estimated in a mouse model of breast cancer by focusing on spatial distributions of iron deposits rather than ROI averages. They found that the polarization status of the iron^+^ populations is affected by contrast-agent injection, which has broad implications for nanoparticle-enhanced biomedical imaging. The paper by K.-H. Jung et al. was focused on the use of new agents for theragnostic approaches based on MRI and neutron capture therapies, showing increased MRI signal in the tumour after therapy. Also, new radiolabelled peptides and antibodies were proposed by M. A. Morcillo et al. as novel PET biomarkers for the diagnosis and prognosis of pancreatic ductal adenocarcinoma. Finally, J. Buck et al. described a novel arterial spin labelling MRI method to perform accurate and robust measurements of cerebral blood flow.

In metabolism and cardiology, different PET and MRI biomarkers were proposed by Y. H. Chung et al. for measuring the myocardial glucose adaptations in high-fat-diet-induced insulin resistance, and novel PET tracers were proposed by S. Hellberg et al. to improve characteristics for imaging atherosclerotic plaque inflammation. In neurology, R. Gandhi et al. reported in their systematic review different uses of preclinical imaging techniques of postischaemia neurovascular remodeling. Finally, a technical research article by D. Deidda et al. proposes a novel PET image reconstruction algorithm by utilizing MRI information. This approach offers the opportunity to extract the time-activity curve from the images so that kinetic information can be calculated without the potential need of continuous arterial blood sampling from animals. All ten articles illustrate that the value of predominantly PET and MRI biomarkers in different animal models offers useful biological information in various clinical applications. It is interesting to note that hybrid PET/MRI imaging was performed only in the technical article, whereas in the others, only PET or MRI was used. Thus, it remains to be seen if this powerful machine can become a mainstream multimodality imaging tool to offer substantially improved information than standalone PET and MRI [7].

We envision the scientific findings and knowledge printed in this special issue demonstrate the importance of PET and MRI imaging biomarkers in preclinical investigations as well as the need for standardisation of the imaging biomarkers of each particular disease model and for the follow-up of new treatments and drugs from the small animal model to the patient.

Finally, we would like to thank the reviewers for their valuable review comments, improving greatly the quality of all submitted papers.
